# Problems of mini-pig breeding

**DOI:** 10.18699/VJ21.032

**Published:** 2021-05

**Authors:** K.S. Shatokhin

**Affiliations:** Novosibirsk State Agrarian University, Novosibirsk, Russia

**Keywords:** aboratory mini-pigs, inbreeding, genetic diversity, recessive mutations, selection, lines, families, agriculture, лабораторные мини-свиньи, инбридинг, генетическое разнообразие, рецессивные мутации, отбор, линии, семейства, сельское хозяйство

## Abstract

This article provides an overview of some problems of the breeding and reproduction of laboratory minipigs. The most obvious of these are the lack of centralized accounting of breeding groups, uniform selection standards for reproduction and evaluation of breeding animals, as well as minimizing the accumulation of fitness-reducing mutations and maintaining genetic diversity. According to the latest estimates, there are at least 30 breeding groups of mini-pigs systematically used as laboratory animals in the world. Among them, there are both breed formations represented by several colonies, and breeding groups consisting of a single herd. It was shown that the main selection strategy is selection for the live weight of adults of 50–80 kg and the adaptation of animals to a specific type of biomedical experiments. For its implementation in the breeding of foreign mini-pigs, selection by live weight is practiced at 140- and 154-day-old age. It was indicated that different herds of mini-pigs have their own breeding methods to counteract inbred depression and maintain genetic diversity. Examples are the maximization of coat color phenotypes, the cyclical system of matching parent pairs, and the structuring of herds into subpopulations. In addition, in the breeding of foreign mini-pigs, molecular genetic methods are used to monitor heterozygosity. Every effort is made to keep the number of inbred crosses in the breeding of laboratory mini-pigs to a minimum, which is not always possible due to their small number. It is estimated that to avoid close inbreeding, the number of breeding groups should be at least 28 individuals, including boars of at least 4 genealogical lines and at least 4 families of sows. The accumulation of genetic cargo in herds of mini-pigs takes place, but the harmful effect is rather the result of erroneous decisions of breeders. Despite the fact that when breeding a number of mini-pigs, the goal was to complete the herds with exclusively white animals, in most breeding groups there is a polymorphism in the phenotype of the coat color

## Abstract (ru)

В статье представлен обзор проблем разведения и селекции лабораторных мини-свиней. Наиболее очевидные из них – отсутствие централизованного учета селекционных групп, единых стандартов отбора для воспроизводства и оценки племенных животных, а также минимизация накопления снижающих приспособленность мутаций и поддержание генетического разнообразия. По последним данным, в мире насчитывают не менее 30 селекционных групп мини-свиней, систематически используемых в качестве лабораторных животных. Среди них существуют как породные образования, представленные несколькими колониями, так и селекционные группы, состоящие из одного стада. Показано, что основная стратегия отбора включает селекцию на живую массу взрослых особей 50–80 кг и приспособленность животных к конкретному типу биомедицинских экспериментов. Для ее реализации в разведении зарубежных мини-свиней практикуют отбор по живой массе в 140- и 154-дневном возрасте. Указано, что в стадах мини-свиней представлены разные селекционные методы противодействия инбредной депрессии и поддержания генетического разнообразия. Примерами служат максимизация фенотипов масти, цикличная система подбора родительских пар и структурирование стад на субпопуляции. Кроме того, в разведении зарубежных мини-свиней для мониторинга гетерозиготности используют молекулярно-генетические методы. Количество инбредных скрещиваний в разведении лабораторных мини-свиней стараются минимизировать, что не всегда возможно из-за их малочисленности. Подсчитано, что во избежание тесного инбридинга численность селекционной группы должна быть не менее 28 особей, включающих хряков как минимум четырех генеалогических линий и свиноматок из не менее четырех семейств. Накопление генетического груза в стадах мини-свиней возможно, но вредоносный эффект является скорее следствием ошибочных решений селекционеров. Несмотря на то что при выведении ряда мини-свиней стояла цель укомплектовать стада исключительно белыми животными, в большинстве селекционных групп наблюдается полиморфизм по фенотипу масти.

## Background

Despite the practicality of laboratory use in comparison with primates and several morphophysiological advantages over other laboratory animals (Tikhonov, 2010; Shatokhin et al., 2019), mini-pigs are still not the most popular biological model, second not only to rodents but also to dogs, cats and monkeys (Heining, Ruysschaert, 2016). However, according to various estimates, there are from 21 to 45 breeding groups of mini-pigs globally (Smith, Swindle, 2006; Köhn, 2011), of which two are bred in Russia (Stankova et al., 2017; Shatokhin et al., 2019). Although, despite the importance of understanding the breeding of any animal species, regardless of their use, the problems of breeding laboratory mini-pigs are shown in a fairly small number of scientific papers. The apparently insufficient attention to the breeding and selection of mini-pigs resulted in some problems and the lack of a unified concept for their solution. The main ones are:

- the lack of centralized accounting of the number of laboratory mini-pigs and the registration system of specialized herds as breeding achievements;
- the lack of generally accepted standards for the selection of animals for reproduction. This also includes the lack of regulatory documents for the evaluation of breeding animals;
- maximizing herds’ genetic diversity under conditions of gene pool depletion vectors (gene drift, bottleneck effect), optimization of monitoring and selection management methods;
- minimizing the accumulation of fitness-reducing mutations;
- the creation of herds of laboratory mini-pigs, staffed exclusively from animals of white coat color;

The purpose of this paper is to describe the listed problems and suggest some ways to solve them.

## The global genetic fund of laboratory mini-pigs

To date, it is difficult to estimate the number of the world’s population of laboratory mini-pigs and the exact number of their breeds, herds, and breeding groups. The main difficulty lies in the absence of a single body for recording laboratory mini-pigs as objects of breeding. For example, according to Russian legislation, the registration of laboratory mini-pigs is difficult due to their formal non-compliance with the criteria for evaluating breeds and breed groups of pigs as breeding achievements, particularly according to the uniformity of the breeding stock (Method of testing for distinctness..., 2007). No special standards have been developed for them. Registration is possible on the website of the American Mini-pig Association (https://americanminipigassociation.com). However, out of 14 registered breeds, only four breeding groups were reliably used as laboratory animals.

The only available accounting tool is scientific publications, but the number of breeding groups of laboratory mini-pigs varies from 21 to 45 (Smith, Swindle, 2006; Tikhonov, 2010). One of the reasons for the discrepancy in the calculation results is the presence of more than one name for the same breed formation. Our own count of laboratory mini-pigs indicated 31 breeding groups in the world (Table 1). Both breed formations are represented by several colonies (Hormel, Hanford, Göttingen, NIH, Yucatan) and breeding groups consisting of a single herd (NIBS; mini-pigs of the Institute of Cytology and Genetics SB RAS, ICG SB RAS; Svetlogorsk). Representatives of the species Sus scrofa L. were taken into account with a live weight of no more than 150 kg and an indication of systematic use as a model object over the past 10 years.

**Table 1.**
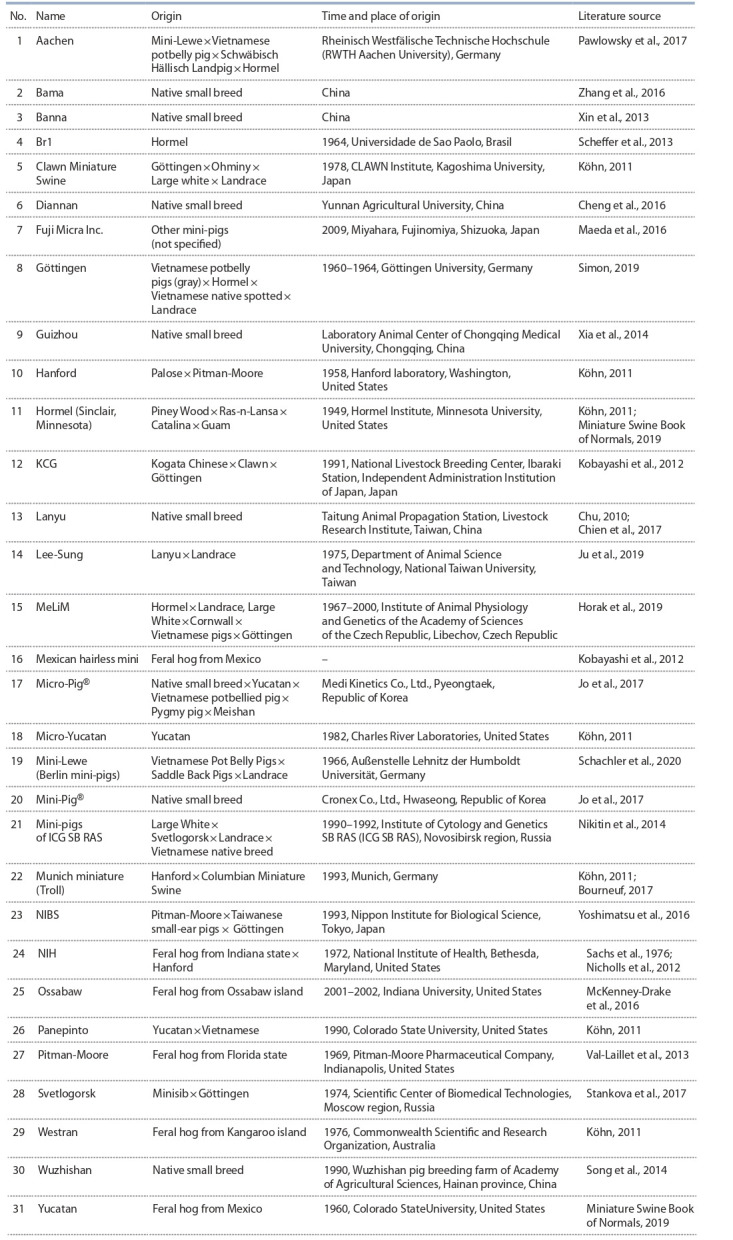
List of the breeding groups of laboratory mini-pigs

## Selection principles of breeding animals

In the breeding of laboratory mini-pigs, there are two main selection vectors: for small size and low live weight and suitability for laboratory use. However, in the breeding of minipigs, there are no uniform specially developed standards for evaluating animals by live weight at an early age, exterior, coat color and a set of characteristics necessary for use in the most common types of biomedical experiments (Helke et al., 2016). Simultaneously, almost every herd has a systematic approach to breeding with its own specific methods (Itoh et al., 2016; Nikitin et al., 2018). Animals are often evaluated at an early age, for example, 140–154 days (Miniature Swine Book of Normals, 2019; Simon, 2019). Some private farms practice selection of the smallest animals from each nest^1^, which in the defunct selection group Minisibs had such consequences as lowering the safety of piglets, sexual activity of boars and destroying the complex of maternal qualities of sows (Nikitin et al., 2014).

Erasmus D. Pigs as pets: Breeding teacup pigs. Farmer’s Weekly. 2013. https://www.farmersweekly.co.za/animals/pigs-as-pets-breeding-teacup-pigs/


The only general principle is selecting the most robust, healthy and proportionally developed animals with a live weight of adults from 50 to 80 kg (Nunoya et al., 2007; Tikhonov2010; Miniature Swine Book of Normals, 2019). Vietnamese mini-pigs’ exterior traits such as a weak back or early obesity are not welcomed by Russian, European and American specialists. Russian mini-pigs and several foreign breeding groups meet the accepted standards, but there are deviations, both in larger and smaller directions (Table 2). Recently, the breeding of herds of tiny pigs weighing 30– 50 kg, for example, German mini-pigs Aachen, American Panepinto and Korean Micro-Pig®, is gaining popularity (see Table 2).

**Table 2.**
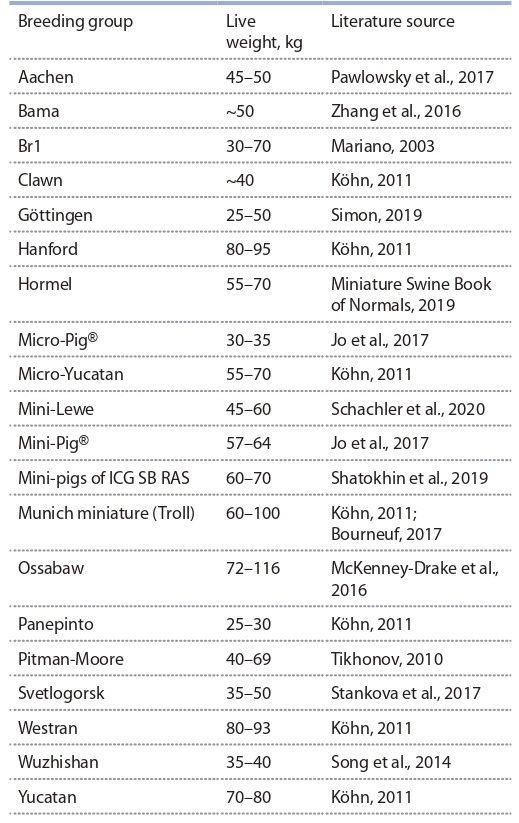
Live weight of adult laboratory mini-pigs from different breeding groups

## The preservation of genetic diversity

The problem of preserving genetic diversity in populations is one of the most discussed issues in animal genetics (Peripolli et al., 2017; Mable, 2019) and, for several reasons, is particularly relevant for laboratory mini-pigs. The first reason is the low population of herds. The risk of depletion of the gene pool due to stochastic processes is significantly higher than in large structured subpopulations communities (Mariani et al., 2020). The second reason is the existence of several breeding groups of laboratory mini-pigs in the singular, which deprives them of such a powerful resource for controlling heterozygosity as the periodic exchange of the gene pool between different herds (Mariani et al., 2020). The third reason is that creating new herds of laboratory mini-pigs from a small number of progenitors (see Table 1) creates a risk of depleting the gene pool due to the bottleneck effect (Ji et al., 2011). Interestingly, according to various estimates, the genetic diversity of laboratory pigs can be both greater and lower compared with similar parameters of pigs of factory breeds and wild boar (Nikitin et al., 2010; Heckel et al., 2015).

Several publications mentioned the existence of natural “contr inbred” mechanisms in natural populations (Charlesworth, Willis, 2009; Cheptou, Donohue, 2011; Mable, 2019), which is indirectly confirmed by the existence of the short populations of feral pigs with no signs of inbreeding depression on small islands throughout the centuries (Köhn, 2011; McKenney-Drake et al., 2016). In the conditions of farms for breeding of laboratory mini-pigs, the formation of the composition of the reproductive group and the choice of parent pairs during the breeding campaign is carried out by the breeder. Therefore, the question about the full functioning of such mechanisms arises. Thus, there is a need to analyze the methods available to humans to control herds’ heterozygosity of laboratory mini-pigs. The first method is monitoring genetic diversity using molecular genetic methods, which is used to select some of the mini-pigs (Chang et al., 2009). A limiting factor in further implementing this method is the lack of data on its economic feasibility in routine use.

The second way to control heterozygosity is to use breeding techniques and methods, for example, to minimize inbred crosses (Simianer, Köhn, 2010). In the breeding of mini-pigs of the ICG SB RAS, the conservation of the maximum possible number of color phenotypes and inbreeding mainly on the progenitors is used to preserve genetic diversity (Nikitin et al., 2018). Given that the mammalian suit is controlled by 120 to 350 genes (Cieslak et al., 2011; Chandramohan et al., 2013), the number of possible genotypes can be in the thousands. Another breeding method for maximizing genetic diversity is dividing an array of animals into subpopulations with a limited gene flow between them (Mariani et al., 2020). However, due to the low number of rock formations, partial genealogical separation of lines, with rare exceptions (Stankova et al., 2017), is practically impossible to implement. Instead, a cyclical selection system is practiced (Chu, 2010; Schachler et al., 2020) based on periodically repeated crosses of lines and families (Table 3). According to the calculations, to avoid close inbreeding, the minimum number of the reproductive group should be at least 28 individuals, of which boars should be represented by at least four lines and sows – by four families. Each line should include at least one main and one checked boar, and the family should consist of at least 5 main and checked sows.

**Table 3.**
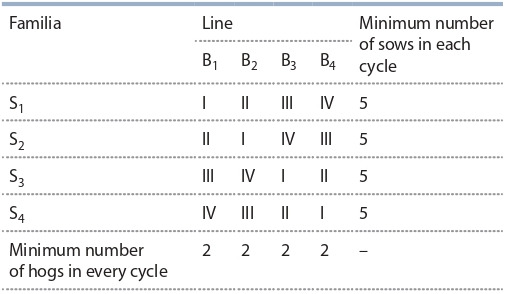
Conditional scheme of the selection of boars and sows during one cycle Notе. The cells at the intersection of lines (columns) and families (rows) indicate the generations of descendants.

## Accumulation of genetic cargo

In the 1970s, it was reported that in populations of less than 2,000 individuals, the probability of accumulation of fitnessreducing mutations is quite high (Nei, Roychoudhury, 1973). Even earlier, it was established that recessive semi-lethal mutations could persist in a population for up to 99 generations even with targeted culling of homozygotes (Dubinin, Glembotsky, 1967), which is generally not refuted by later mathematical modelling (Johnsson et al., 2019). It is considered that the elimination of harmful recessive mutations is a difficult task for the breeder, even if he uses modern genotyping methods (Derks et al., 2017). Given that the reproductive number of individual herds of laboratory mini-pigs does not exceed 30– 40 individuals reducing sustainability, semi-lethal and lethal recessive mutations pose a danger in breeding these animals. At the same time, in the entire history of breeding laboratory mini-pigs, only in the extinct breeding group Minisibs a decrease in the viability of young animals and the reproductive qualities of adults was described, the alleged cause of which was the accumulation of recessive mutations due to unilateral selection (Nikitin et al., 2014). Thus, laboratory mini-pigs’ breeding system should include measures to purify the herd from harmful mutations, leading to strict selection in the reproductive group (Nikitin et al., 2018, 2020). Another method of cleaning herds from unwanted mutations is to assess the progeny in the inbred cross. This method was proposed for various farm animals’ species in the 1950s and 1970s (Robertson, Rendel, 1950; Serebrovsky, 1970). However, despite its simplicity, the method has a serious drawback – it is the duration of the assessment and, accordingly, the cost of feeding and maintaining the tested boar and its descendants.

However, there are cases where breeders have benefited from the emergence of viability-reducing mutations in the herd in the form of creating model objects to optimize specific medical methods or treat strictly defined pathologies. Examples are the creation of mini-pigs by MeLiM and NIH (Sachs et al., 1976; Horak et al., 2019). Thus, it can be argued that the very fact of the occurrence of mutations that reduce viability, of course, is a danger. But much more important is breeders’ ability to prioritize the selection of animals for reproduction and to carry out measures to clear the herds of genetic cargo; and if necessary, to consolidate the carriers of mutations in the form of a new selection group that is of value as a model object.

## The problem of white coat color in the breeding of laboratory mini-pigs

It is known that when creating the first breeding groups of laboratory mini-pigs, the task was to create white-colored animals (Pond, Houpt, 1978), which were planned to be used as a biological model for studying the effects of radioactive radiation on the skin. However, despite the “influx of blood” of factory breeds of white color, attempts to consolidate it in herds of laboratory mini-pigs, as a rule, did not succeed. The exceptions are the Mini-Lewe pigs (Schachler et al., 2020) and the Bintang line (Lanyu 400) in the Lanyu mini-pig breeding group (Chu, 2010), but most herds have polymorphism by suit type (Mariano, 2003; Tikhonov, 2010; https://ameri canminipigassociation.com). Thus, the question arises about the factors that prevent the breeding of herds fully equipped with white individuals. It can be assumed that this is due to the dominant control of the most common type of white coat color (Pielberg et al., 2002), which is why there is a regular cleavage of pigmented piglets. Another explanation is that white piglets are born smaller and, therefore, less viable than colored animals (Nikitin et al., 2019). Despite this, the white coat color was successfully consolidated in a factory breeds series (Porter et al., 2016). It should be noted that the factory breeds of white-colored pigs were obtained by the method of more than 70 years of selection of white individuals in each generation with a preference for those animals in whose offspring there was no splitting according to the color phenotype (Porter et al., 2016). And this, in turn, is comparable to the duration of the oldest breeding groups of laboratory mini-pigs (Tikhonov, 2010). Thus, it can be assumed that the breeders of most breeding groups of mini-pigs simply did not have enough time to consolidate the white suit.

Molecular genetic typing of white animals would significantly speed up the process of fixing the white suit. It is known that the dominant white color of pigs is controlled by allele I of the KIT gene (Pielberg et al., 2002; Wu et al., 2019). Thus, the first step to create a breeding group complete with all-white animals should be to cross white sows with white boars. All-white offspring from such crosses will need to be genotyped according to the KIT gene with the setting of homozygotes (I/I ) for rearing. The method of determining the KIT gene’s alleles using real-time PCR is described in detail in the literature (Pielberg et al., 2002).

Another way is to consolidate the recessive white suit’s phenotype, as demonstrated by the Lanyu 400 line (Chi, 2010) and the Chinese Rongchang breed (Lai et al., 2007). However, a rather serious restriction on using this method may be the low frequency of cleavage of recessive white color individuals, which in the herd of mini-pigs of the Institute of Cytology and Genetics SB RAS, according to zootechnical accounting, is about 1 %.

## Conclusion

Over the past 10 years, facts have been discovered confirming the existence of 31 breeding groups of mini-pigs. Despite the lack of uniform selection standards in breeding laboratory mini-pigs, they adhere to such general criteria as a live weight of 50–80 kg, normal viability, and the strength of the animals’ constitution and exterior. Maintaining genetic diversity in herds of laboratory mini-pigs is possible both with the use of molecular genetic monitoring and purely selective methods. The minimization of the negative effect of genetic cargo accumulation in the herds of mini-pigs should be implemented mainly through a strict selection for fitness in the reproductive group. If necessary, due to the need for a specific type of biomedical experiments, it is possible to fix external and physiological characteristics in the herd, controlled by recessive mutations that reduce viability. Consolidation of white individuals is possible, which is proved by the examples of the Bintang line and the Mini-Lewe breeding group.

## Conflict of interest

The authors declare no conflict of interest.
